# Increased Alveolar Epithelial Damage Markers and Inflammasome-Regulated Cytokines Are Associated with Pulmonary Superinfection in ARDS

**DOI:** 10.3390/jcm12113649

**Published:** 2023-05-24

**Authors:** Konrad Peukert, Andrea Sauer, Benjamin Seeliger, Caroline Feuerborn, Mario Fox, Susanne Schulz, Lennart Wild, Valeri Borger, Patrick Schuss, Matthias Schneider, Erdem Güresir, Mark Coburn, Christian Putensen, Christoph Wilhelm, Christian Bode

**Affiliations:** 1 Department of Anesthesiology and Intensive Care Medicine, University Hospital Bonn, Venusberg-Campus 1, 53127 Bonn, Germany; 2 Department of Respiratory Medicine and German Centre of Lung Research (DZL), Hannover Medical School, Carl-Neuberg-Str. 1, 30635 Hannover, Germany; 3 Department of Neurosurgery, University Hospital Bonn, Venusberg-Campus 1, 53127 Bonn, Germany; 4 Department of Neurosurgery, BG Klinikum Unfallkrankenhaus Berlin gGmbH, Warener Str. 7, 12683 Berlin, Germany; 5 Department of Neurosurgery, University Hospital Leipzig, Liebig Str. 20, Haus 4, 04103 Leipzig, Germany; 6 Institute of Clinical Chemistry and Clinical Pharmacology, University Hospital Bonn, Venusberg-Campus 1, 53127 Bonn, Germany

**Keywords:** pulmonary superinfection, inflammasome, molecular phenotyping, acute respiratory distress syndrome, precision medicine, pneumonia, influenza

## Abstract

Acute respiratory distress syndrome (ARDS) is a life-threatening form of respiratory failure defined by dysregulated immune homeostasis and alveolar epithelial and endothelial damage. Up to 40% of ARDS patients develop pulmonary superinfections, contributing to poor prognosis and increasing mortality. Understanding what renders ARDS patients highly susceptible to pulmonary superinfections is therefore essential. We hypothesized that ARDS patients who develop pulmonary superinfections display a distinct pulmonary injury and pro-inflammatory response pattern. Serum and BALF samples from 52 patients were collected simultaneously within 24 h of ARDS onset. The incidence of pulmonary superinfections was determined retrospectively, and the patients were classified accordingly. Serum concentrations of the epithelial markers soluble receptor for advanced glycation end-products (sRAGE) and surfactant protein D (SP-D) and the endothelial markers vascular endothelial growth factor (VEGF) and angiopoetin-2 (Ang-2) as well as bronchoalveolar lavage fluid concentrations of the pro-inflammatory cytokines interleukin 1ß (IL-1ß), interleukin 18 (IL-18), interleukin 6 (IL-6), and tumor necrosis factor-alpha (TNF-a) were analyzed via multiplex immunoassay. Inflammasome-regulated cytokine IL-18 and the epithelial damage markers SP-D and sRAGE were significantly increased in ARDS patients who developed pulmonary superinfections. In contrast, endothelial markers and inflammasome-independent cytokines did not differ between the groups. The current findings reveal a distinct biomarker pattern that indicates inflammasome activation and alveolar epithelial injury. This pattern may potentially be used in future studies to identify high-risk patients, enabling targeted preventive strategies and personalized treatment approaches.

## 1. Introduction

Acute respiratory distress syndrome (ARDS) is a heterogeneous syndrome characterized by a dysregulated inflammatory host response leading to severe alveolar epithelial and endothelial injury. A subsequent loss of alveolar–capillary barrier integrity results in the accumulation of protein-rich edema fluid in the lung interstitium and critical arterial hypoxemia in ARDS patients [1]. One of the main complications of ARDS is the development of pulmonary superinfections contributing to negative outcomes and excess mortality. Up to 40% of patients suffering from ARDS develop pulmonary superinfections over the course of treatment [2,3]. Major risk factors include a loss of epithelial barrier function, prolonged mechanical ventilation, and prone positioning which might facilitate microbial dissemination and increase the risk for abundant microaspiration of gastric contents. Pulmonary dysbiosis in combination with defects in innate and adaptive immunity may further explain the high incidence of pulmonary superinfection [4,5,6]. Hence, it is imperative to understand what predisposes ARDS patients to pulmonary superinfections to tailor future clinical trials and to be able to adjust treatment accordingly.

Several biomarkers indicating lung endothelial and epithelial damage as well as pulmonary inflammation in ARDS patients have been identified so far and are promising tools to refine molecular phenotyping, assess prognosis, and evaluate treatment response [7,8]. Although biomarkers have been validated for ARDS, little is known about their predictive value for pulmonary superinfections in ARDS.

Serum surfactant protein D (SP-D) and soluble receptor for advanced glycation end-products (sRAGE) are both promising markers of alveolar epithelial injury which have both been linked to poor prognosis in ARDS [9,10,11]. Furthermore, external validation of biomarkers and a clinical prediction model for hospital mortality in ARDS patients included SP-D in a variety of clinical settings and may be useful in risk assessments for clinical trial enrolment [12]. In contrast, vascular endothelial growth factor (VEGF) and Angiopoetin-2 (Ang-2) reflect endothelial injury in ARDS [13,14] and predict ARDS onset as well as increased mortality [15,16].

Lung injury including epithelial and endothelial damage is mediated by inflammatory cytokines [1]. In particular, inflammasome activation and its downstream cytokines IL-1ß and IL-18 are major contributors to lung injury in ARDS and correlate with an unfavorable outcome [17,18,19,20]. Inflammasomes are pivotal components of the innate immune system that consist of a sensor NOD-, LRR-, and pyrin-domain-containing protein 3 (NLRP3), an adaptor-apoptosis-associated speck-like protein containing a CARD (ASC), and an effector (caspase-1) [8,21]. Its activation is tightly controlled by a two-step mechanism. Step one or the priming signal is initiated by pattern recognition receptors (PRRs) such as TLRs (toll-like receptors) that sense a diverse set of microbial molecules, termed pathogen-associated molecular patterns (PAMPs), such as bacterial lipopolysaccharides (LPS) or endogenous damage-associated molecular patterns (DAMPs) including ATP, mitochondrial DNA, and fibrinogen. As a result of, e.g., TLR-4 sensing LPS, transcription factor nuclear factor kappa B (NF-κb) becomes activated leading to the subsequent upregulation of the sensor NLRP3 and pro-interleukin-1ß (pro-IL-1ß). A plethora of stimuli including extracellular ATP, pathogen-associated RNA, and bacterial pore-forming toxins can activate NLRP3, triggering inflammasome assembly via the recruitment of adaptor protein ASC (step two). The assembled inflammasome includes activated caspase 1 which cleaves pro-IL-1ß and pro-IL-18 into their biologically active forms IL-1ß and IL-18, inducing pyroptosis, a form of alternative inflammatory cell death [8,22,23]. Excess inflammation and deleterious pyroptosis are major drivers of pulmonary injury and may predispose ARDS patients to pulmonary superinfections [1,5,24].

We hypothesize that ARDS patients who develop pulmonary superinfections exhibit a distinct pulmonary injury and inflammatory response pattern. To test this hypothesis, we analyzed epithelial and endothelial damage markers as well as pro-inflammatory markers in ARDS patients with and without pulmonary superinfections.

## 2. Materials and Methods

### 2.1. Study Design and Population

We performed a single-center, retrospective analysis of ARDS patients hospitalized at the University Hospital Bonn, Bonn, Germany. ARDS was diagnosed according to the Berlin definition of the 2012 announcement which defines ARDS as the acute onset of hypoxemia with bilateral infiltrates and no evidence of left atrial hypertension [25]. Bronchoalveolar lavage fluid (BALF) and serum samples were collected within 24 h of disease onset. The incidence of pulmonary superinfection was then determined in a retrospective analysis via electronic health records.

If ARDS was already present at the time of hospital admission, we defined ARDS onset as the time of symptom onset. Pulmonary superinfection was defined as any secondary pulmonary infection caused by bacterial, viral, or fungal pathogens that occurred within 28 days after ARDS onset. Diagnosis of pulmonary superinfection was confirmed by pathogen detection in microbial cultures or via RT-PCR accompanied by increased secondary white cell count and procalcitonin (PCT) as well as the presence of new or progressive pulmonary infiltrates on chest radiographs or chest computed tomography (CT) scans.

BALF and serum samples were obtained from ARDS patients of the University Hospital Bonn, Bonn, Germany, with approval of the Institutional Review Board of the University Hospital Bonn, Bonn, Germany (No.088/16). The study was conducted according to the guidelines of the Declaration of Helsinki. Written informed consent was obtained from all participants prior to inclusion in this study.

### 2.2. Sample Collection and Processing

BALF and serum samples were collected simultaneously within 24 h of ARDS onset. Blood samples were collected from ARDS patients using serum gel monovettes (S-Monovette, Sarstedt AG & Co. KG, Nuembrecht, Germany). After centrifugation at 2500× *g* and room temperature for 10 min, the serum samples were aliquoted into cryotubes and stored at −80 °C until further processing.

A standard bronchoscopy protocol was used to obtain BALF for bacterial and virological testing as described before [8]. In brief, bronchoalveolar lavage (BAL) was performed with a flexible bronchoscope wedged in a segment of the right middle lobe. A total quantity of 200 mL of normal saline was instilled in 4 aliquots with a 50 mL syringe and added tubing, and BALF was recovered by manual aspiration. The BALF samples were immediately placed on ice after collection and centrifuged at 400× *g* and 4 °C for 5 min. The supernatant was stored at −80 °C until further processing. Levels of SP-D, RAGE, Ang-2, VEGF, IL-18, IL-1ß, TNF-α, and IL-6 were analyzed by multiplex immunoassay (Luminex Assay, Bio-Techne, Minneapolis, MN, USA).

### 2.3. Data Collection

The data collected from electronic health records included patient demographics (age, gender), epidemiology, comorbidities, physiological and laboratory parameters (white cell count, procalcitonin, and blood gas analysis), including those used to calculate the Sequential Organ Failure Assessment (SOFA) score, microbiology and virology results (respiratory tract cultures and viral RT-PCRs), ventilatory parameters, immunosuppressive medication administration, and short- and long-term outcomes. The electronic health records of all ARDS patients included in this study were reviewed by several researchers to ensure clinical significance.

### 2.4. Statistical Analysis

The statistical analysis and calculations were performed with GraphPad Prism Software (Version 9.0, La Jolla, CA, USA); a *p*-value < 0.05 was considered statistically significant. The patient characteristics were compared by the Kruskal–Wallis test or Fisher’s exact test and expressed as median, 25% percentile, and 75% percentile. For better comparability and to achieve normal distribution, the biomarker data were log-transformed and presented as an individual value with mean ± SD. Comparisons between groups were analyzed by an unpaired *t*-test.

## 3. Results

### 3.1. Patient Characteristics

Fifty-two ARDS patients who were treated at the University Hospital Bonn, Bonn, Germany, from October 2018 until October 2020 were included in this retrospective study. A total quantity of 25 ARDS patients developed pulmonary superinfections over the course of treatment. The main characteristics of the ARDS patients with and without superinfections are shown in Table 1 and Table S1. No significant differences in demographics, comorbidities, immunocompromised conditions, ventilatory settings, inflammatory parameters, disease severity, and mortality were observed between the ARDS patients that developed or did not show pulmonary superinfections.

### 3.2. Epithelial Damage Markers Differ in ARDS Patients with and without Secondary Pulmonary Infection

**Figure 1:**
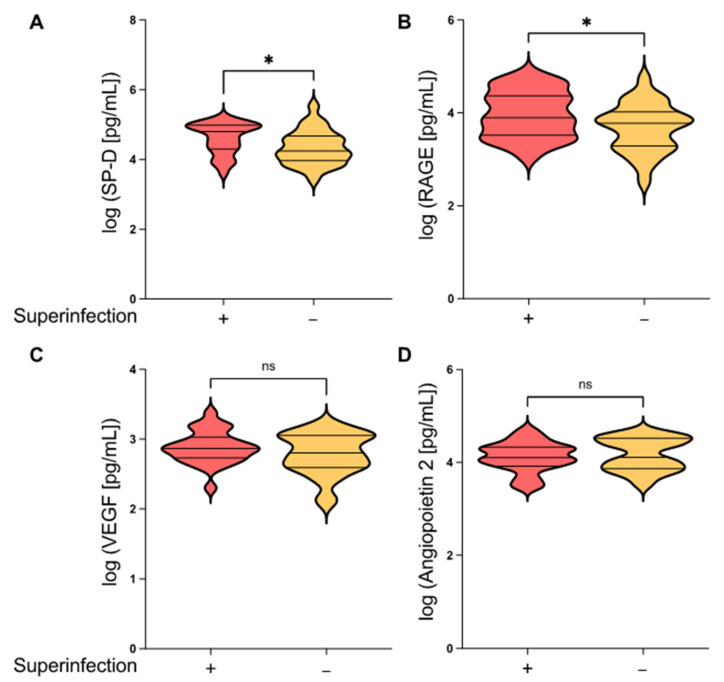
Epithelial damage markers are differentially expressed in ARDS patients with and without pulmonary superinfections. Violin plots of (**A**) SP-D, (**B**) sRage, (**C**) VEGF, and (**D**) Angiopoetin-2 serum levels in ARDS patients with and without pulmonary superinfections. Blood samples were collected from ARDS patients within 24 h of disease onset and analyzed via multiplex immunoassay. A total of 25 ARDS patients with pulmonary superinfections and 27 ARDS patients without pulmonary superinfections were included in this study. Mean ± SD of log-transformed data; unpaired *t*-test; * *p* ≤ 0.05; ns = not significant; red violin plot = ARDS with superinfection; yellow violin plot = ARDS without superinfection.

Epithelial barrier function plays an important role in preventing superinfections. Both sRAGE and SP-D have previously been described as promising biomarkers to assess epithelial damage in ARDS patients and are associated with an unfavorable prognosis in ARDS patients [9,10,11]. To investigate whether these lung epithelial damage markers are also associated with pulmonary superinfections in ARDS patients, we determined the levels of sRAGE and SP-D in serum samples drawn within 24 h of ARDS onset. As shown in Figure 1A, the serum concentrations of SP-D and sRAGE were both significantly increased in ARDS patients with pulmonary superinfections compared to ARDS patients who did not develop pulmonary superinfections (*p* = 0.0397 and *p* = 0.0495, respectively). We next tested whether endothelial damage might also be associated with pulmonary superinfections. Ang-2 and VEGF both play a central role in activating endothelial cells and increasing microvascular permeability. We therefore monitored the endothelial injury molecules Ang-2 and VEGF and did not observe any differences in serum levels between ARDS patients with and without pulmonary superinfections. Altogether, we found that epithelial rather than endothelial damage markers were increased in patients with secondary infections.

### 3.3. Inflammasome-Regulated Cytokines Differ in ARDS Patients with and without Secondary Pulmonary Infection

**Figure 2:**
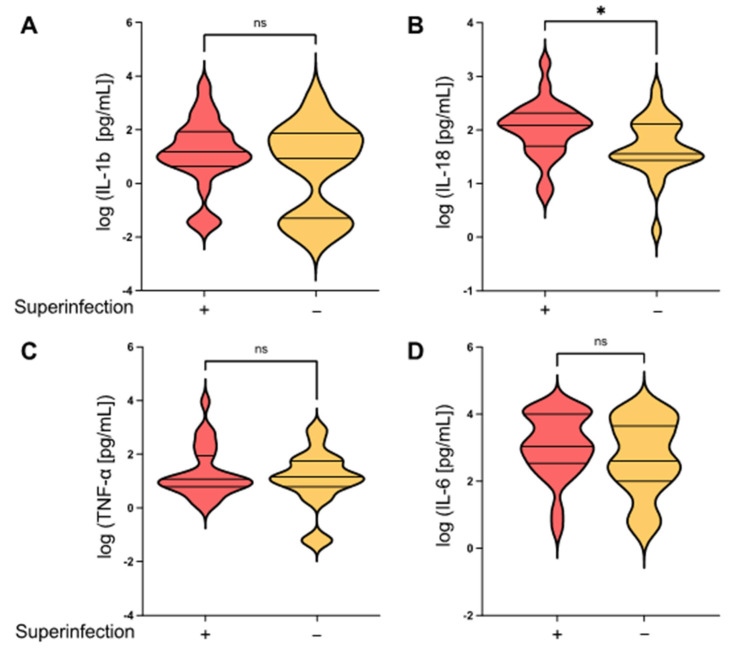
Pulmonary superinfections in patients with ARDS are associated with the increased production of inflammasome-dependent cytokines. Flexible fiberoptic bronchoscopy was performed within 24 h of disease onset. Bronchoalveolar lavage fluid (BALF) samples were collected in the right middle lobe and analyzed afterward via multiplex immunoassay. BALF concentrations of (**A**) IL-1ß, (**B**) IL-18, (**C**) TNF-α, and (**D**) IL-6 compared between ARDS patients with and without pulmonary superinfections. Mean ± SD of log-transformed data; unpaired *t*-test; * *p* ≤ 0.05; ns = not significant; red violin plot = ARDS with superinfection; yellow violin plot = ARDS without superinfection.

As inflammation is a modulator of host susceptibility to pulmonary superinfections [5], we investigated the local pro-inflammatory cytokine milieu in the lungs. In particular, the inflammasome-regulated cytokines IL-1ß and IL-18 are crucial mediators of pulmonary hyperinflammation in ARDS [8,17]. We therefore determined the BALF levels of IL-1ß and IL-18 as well as TNF-α and IL-6 which are known to be elevated in ARDS patients with fatal outcomes [11,20]. Interestingly, the inflammasome-regulated cytokine IL-18 (but not IL-1ß) was significantly increased in ARDS patients who developed pulmonary superinfections compared to patients without a secondary infection (*p* = 0.0271). In contrast, no significant differences between the groups were detected for the inflammasome-independent mediators TNF-α and IL-6 (Figure 2).

## 4. Discussion

Pulmonary superinfection significantly influences patients’ outcomes. Although the pathogenesis of ARDS development is well studied, the underlying mechanisms of the development of pulmonary superinfection remain not well understood. Besides the loss of epithelial barrier function, prolonged mechanical ventilation, and prone positioning, pulmonary dysbiosis in combination with altered immune defenses are major risk factors [4,5,6].

In this study, we showed that the alveolar epithelial damage markers sRAGE and SP-D were significantly increased in ARDS patients who developed pulmonary superinfections while the levels of the endothelial injury markers VEGF and Ang-2 did not differ between the groups (Figure 1). Furthermore, ARDS patients with pulmonary superinfections demonstrated increased levels of inflammasome-regulated IL-18 but not Il-1ß (Figure 2B).

To the best of our knowledge, the current study is the first report that links alveolar epithelial damage markers in ARDS to pulmonary superinfection. Yet, whether sRAGE and SP-D are direct injurious mediators or just markers of tissue damage that lead to pulmonary superinfection is unknown. sRAGE and SP-D are both elevated in ARDS patients and correlate with increased mortality and the severity of disease [9,26,27,28]. Patients with pneumonia or ARDS caused by influenza A virus infection are highly susceptible to co-infections and are characterized by increased SP-D and sRAGE levels [4,11,29,30,31,32]. This might be explained by multifactorial pathogenesis. Elevated levels of SP-D and sRAGE may indicate a loss of barrier function rendering patients more susceptible to pulmonary superinfections by forming new bacterial attachment sites and allowing bacterial translocation [6]. Furthermore, SP-D strongly potentiates the neutrophil respiratory burst in the presence of the influenza A virus by increasing the neutrophil uptake of the influenza A virus [33]. Similar to SP-D, sRAGE has been shown to promote a pro-inflammatory response by activating Nf-Κb. A side effect of RAGE signaling is the induction of reactive oxygen species (ROS), which can also activate Nf-Κb and boost other pro-inflammatory pathways such as cellular apoptosis [34]. Nf-Kb and ROS serve as key inflammasome activators triggering inflammasome assembly which mediates caspase-1 activation and subsequently the release of pro-inflammatory IL-1ß and IL-18 [21].

Inflammasome activation and consecutive IL-1ß and IL-18 production play a major role in the development of ARDS by driving tissue inflammation and a rapid, pro-inflammatory form of cell death called pyroptosis [8,17,35,36]. The subsequent loss of pulmonary epithelial cells might lead to immune barrier dysfunction, thereby increasing the susceptibility to pulmonary superinfections [37]. Accordingly, we observed significantly increased IL-18 concentrations and lung epithelial damage in ARDS patients who developed pulmonary superinfections in comparison to patients without pulmonary superinfections. Consistent with this, murine studies suggest that the production of inflammasome-regulated cytokines including IL-18 may contribute to the increased susceptibility to pulmonary superinfections [24,38,39]. Furthermore, inflammasome adaptor ASC^−/−^ mice possessing a dysfunctional inflammasome function were protected from bacterial superinfection and associated lethality [40]. Yet, the current study found no differences in IL-1ß concentrations between ARDS patients with and without pulmonary superinfections. This might be explained by the extremely short half-life of IL-1ß, which is therefore often undetectable even in human pathologies that are clearly mediated by IL-1ß [40,41,42,43]. In addition, the immunopathological activity of IL-1β in ARDS patients might also be confined to local secretion and paracrine signaling that cannot be captured by a universal detection method such as BAL [43,44,45]. Therefore, previous studies that investigated the role of inflammasome activation in ARDS also focused on IL-18 production as a readout [17,46].

This study has several limitations, primarily its small sample size and single-center status which do not allow us to draw far-reaching conclusions from the results. The data from this study should be regarded as hypothesis-generating and used to design future confirmatory trials with larger cohorts which may allow us to define cut-off values for pulmonary superinfection biomarkers. Due to the retrospective nature of this study, routine systematic testing for pulmonary superinfection was not performed. Moreover, the decision to obtain microbiological and virological testing depended heavily on the treating physician and disease severity of the patient which might have indirectly created selection bias. The sensitivity of quantitative BAL cultures is as high as 90% for the diagnosis of bacterial infection and up to 80% in mycobacterial, fungal, and most viral infections [47]. However, false negative rates vary among studies possibly due to the lack of a uniform threshold for positive BAL cultures. The use of RT-PCR was also mostly limited to virological testing while multiplex PCR, which has shown superior sensitivity for the detection of respiratory lower tract infections compared to quantitative bacterial cultures [48], has rarely been performed. Hence, the number of pulmonary superinfections may be underestimated in our study. Another limitation is the lack of a universal, valid definition of pulmonary superinfection making it a challenging diagnosis. Timing, chest imaging, laboratory values, and microbiology and/or virology results in conjunction with clinical parameters should be incorporated into the definition. Lastly, the complexity and host susceptibility for pulmonary superinfection cannot be fully mirrored by biomarkers measured in serum and BALF at one specific time point. Although the identification of a unique time point can be easily implemented into clinical trials and routine practice, it may not fully reflect the intricate release kinetics of each individual biomarker, thus suggesting the superiority of serial measurements vs. single measurements. Serial measurements at the time of ICU admission and over the course of treatment may provide better prognostic information and ensure reliability. In addition, SP-D, sRAGE, and Ang-2, for example, are biomarkers that display high sensitivity and specificity for the diagnosis or outcome prediction of ARDS but have not been evaluated for pulmonary superinfections in ARDS patients [7].

## 5. Conclusions

In summary, the current study demonstrates that ARDS patients who develop pulmonary superinfections may exhibit a distinct biomarker pattern that indicates epithelial injury and inflammasome activation upon ICU admission. Our findings raise the question of whether this biomarker pattern could potentially be utilized to identify high-risk patients, possibly implementing targeted prevention and facilitating personalized treatment approaches.

## Figures and Tables

**Table 1:** Characteristics of ARDS patients with and without pulmonary superinfections.

Characteristics	ARDS (Superinfection) (*n* = 25)	ARDS (No Superinfection) (*n* = 27)	*p*
Age (y)	60 (45–69)	53 (44–58)	0.158
Male (%)	68	85	0.1933
BMI (kg/m^2^)	31.1 (27–37.6)	29.39 (27.2–34.1)	0.4315
Diabetes (%)	12	18.5	0.705
Immunosuppression (%)	8	7.4	>0.9999
Steroids (%)	20	37	0.2274
PaO_2_/FiO_2_ ratio (mmHg)	80 (68.5–116.5)	92 (64.9–161.5)	0.4642
PEEP (cmH_2_O)	19 (15–20)	18 (15–20)	0.7482
Driving pressure (cmH_2_O)	10 (7.5–13.5)	9 (6–12)	0.33
Tidal volume (ml/kg predicted body weight)	2.5 1.7–4.1	3.3 (1.9–6.4)	0.3555
Procalcitonin (µg/L)	17.1 (1.4–43,6)	5.57 (1.2–45.8)	0.7714
Lactate (mmol/L)	1.9 (1.6–4.6)	1.68 (1.2–3.1)	0.5276
SOFA score (best assumed)	8 (7–10.5)	8 (6–11)	0.6636
ICU mortality (%)	36	33.3	>0.9999

The data are presented as median, 25% percentile, and 75% percentile using the Kruskall–Wallis test or Fisher’s exact test. Abbreviations: BMI, body mass index; PEEP, positive end-expiratory pressure; SOFA score, sepsis-related organ failure assessment score (best assumed for CNS).

## Data Availability

The study materials and datasets are available from the corresponding author upon reasonable request.

## Supplementary Materials

The following supporting information can be downloaded at: https://www.mdpi.com/article/10.3390/jcm12113649/s1. Table S1: Origin of ARDS in patients with and without pulmonary superinfection.

## References

[1] Matthay M.A., Zemans R.L., Zimmerman G.A., Arabi Y.M., Beitler J.R., Mercat A., Herridge M., Randolph A.G., Calfee C.S. (2019). Acute respiratory distress syndrome. Nat Rev Dis Primer. Nat. Publ. Group.

[2] Ayzac L., Girard R., Baboi L., Beuret P., Rabilloud M., Richard J.C., Guérin C. (2016). Ventilator-associated pneumonia in ARDS patients: The impact of prone positioning. A secondary analysis of the PROSEVA trial. Intensive Care Med..

[3] Forel J.-M., Voillet F., Pulina D., Gacouin A., Perrin G., Barrau K., Jaber S., Arnal J.-M., Fathallah M., Auquier P. (2012). Ventilator-associated pneumonia and ICU mortality in severe ARDS patients ventilated according to a lung-protective strategy. Crit. Care.

[4] Luyt C.-E., Bouadma L., Morris A.C., Dhanani J.A., Kollef M., Lipman J., Martin-Loeches I., Nseir S., Ranzani O.T., Roquilly A. (2020). Pulmonary infections complicating ARDS. Intensive Care Med..

[5] Aguilera E.R., Lenz L.L. (2020). Inflammation as a Modulator of Host Susceptibility to Pulmonary Influenza, Pneumococcal, and Co-Infections. Front. Immunol..

[6] Paget C., Trottein F. (2019). Mechanisms of Bacterial Superinfection Post-influenza: A Role for Unconventional T Cells. Front. Immunol..

[7] Spadaro S., Park M., Turrini C., Tunstall T., Thwaites R., Mauri T., Ragazzi R., Ruggeri P., Hansel T.T., Caramori G. (2019). Biomarkers for Acute Respiratory Distress syndrome and prospects for personalised medicine. J. Inflamm..

[8] Peukert K., Fox M., Schulz S., Feuerborn C., Frede S., Putensen C., Wrigge H., Kümmerer B.M., David S., Seeliger B. (2021). Inhibition of Caspase-1 with Tetracycline Ameliorates Acute Lung Injury. Am. J. Respir. Crit. Care Med..

[9] Jabaudon M., Blondonnet R., Pereira B., Cartin-Ceba R., Lichtenstern C., Mauri T., Determann R.M., Drabek T., Hubmayr R.D., Gajic O. (2018). Plasma sRAGE is independently associated with increased mortality in ARDS: A meta-analysis of individual patient data. Intensive Care Med..

[10] Eisner M., Parsons P., Matthay M., Ware L., Greene K. (2003). Plasma surfactant protein levels and clinical outcomes in patients with acute lung injury. Thorax.

[11] Peukert K., Seeliger B., Fox M., Feuerborn C., Sauer A., Schuss P., Schneider M., David S., Welte T., Putensen C. (2021). SP-D Serum Levels Reveal Distinct Epithelial Damage in Direct Human ARDS. J. Clin. Med..

[12] Zhao Z., Wickersham N., Kangelaris K.N., May A.K., Bernard G.R., Matthay M.A., Calfee C.S., Koyama T., Ware L.B. (2017). External validation of a biomarker and clinical prediction model for hospital mortality in acute respiratory distress syndrome. Intensiv. Care Med..

[13] Fremont R.D., Koyama T., Calfee C.S., Wu W., Dossett L.A., Bossert F.R., Mitchell D., Wickersham N., Bernard G.R., Matthay M.A. (2010). Acute Lung Injury in Patients with Traumatic Injuries: Utility of a Panel of Biomarkers for Diagnosis and Pathogenesis. J. Trauma.

[14] Terpstra M.L., Aman J., van Nieuw Amerongen G.P., Groeneveld A.B.J. (2014). Plasma biomarkers for acute respiratory distress syndrome: A systematic review and meta-analysis. Crit Care Med..

[15] Agrawal A., Matthay M.A., Kangelaris K.N., Stein J., Chu J.C., Imp B.M., Cortez A., Abbott J., Liu K.D., Calfee C.S. (2013). Plasma angiopoietin-2 predicts the onset of acute lung injury in critically ill patients. Am. J. Respir. Crit. Care Med..

[16] Wada T., Jesmin S., Gando S., Yanagida Y., Mizugaki A., Sultana S.N., Zaedi S., Yokota H. (2013). The role of angiogenic factors and their soluble receptors in acute lung injury (ALI)/acute respiratory distress syndrome (ARDS) associated with critical illness. J. Inflamm..

[17] Dolinay T., Kim Y.S., Howrylak J., Hunninghake G.M., An C.H., Fredenburgh L., Massaro A.F., Rogers A., Gazourian L., Nakahira K. (2012). Inflammasome-regulated cytokines are critical mediators of acute lung injury. Am. J. Respir. Crit. Care Med..

[18] RRogers A.J., Guan J., Trtchounian A., Hunninghake G.M., Kaimal R., Desai M., Kozikowski L.-A., DeSouza L., Mogan S., Liu K.D. (2019). Association of Elevated Plasma Interleukin-18 Level With Increased Mortality in a Clinical Trial of Statin Treatment for Acute Respiratory Distress Syndrome. Crit. Care Med..

[19] Grailer J.J., Canning B.A., Kalbitz M., Haggadone M.D., Dhond R.M., Andjelkovic A.V., Zetoune F.S., Ward P.A. (2014). Critical role for the NLRP3 inflammasome during acute lung injury. J. Immunol..

[20] Meduri G.U., Kohler G., Headley S., Tolley E., Stentz F., Postlethwaite A. (1995). Inflammatory cytokines in the BAL of patients with ARDS. Persistent elevation over time predicts poor outcome. Chest.

[21] McVey M.J., Steinberg B.E., Goldenberg N.M. (2021). Inflammasome activation in acute lung injury. Am. J. Physiol. Lung Cell Mol. Physiol. Am. Physiol. Soc..

[22] Guo H., Callaway J.B., Ting J.P.-Y. (2015). Inflammasomes: Mechanism of action, role in disease, and therapeutics. Nat. Med..

[23] Hornung V., Latz E. (2010). Critical functions of priming and lysosomal damage for NLRP3 activation. Eur. J. Immunol..

[24] Robinson K.M., Ramanan K., Clay M., McHugh K.J., Pilewski M.J., Nickolich K.L., Corey C., Shiva S., Wang J., Alcorn J.F. (2018). The inflammasome potentiates influenza/Staphylococcus aureus superinfection in mice. J. Clin. Investig..

[25] Force A.D.T., Ranieri V.M., Rubenfeld G.D., Thompson B., Ferguson N., Caldwell E., Fan E., Camporota L., Slutsky A.S. (2012). The ARDS Definition Task Force. Acute Respiratory Distress Syndrome: The Berlin Definition. JAMA.

[26] Agustama A., Surgean Veterini A., Utariani A. (2022). Correlation of Surfactant Protein-D (SP-D) Serum Levels with ARDS Severity and Mortality in Covid-19 Patients in Indonesia. Acta Medica Acad..

[27] Dahmer M.K., Flori H., Sapru A., Kohne J., Weeks H.M., Curley M.A., Matthay M.A., Quasney M.W., Bateman S.T., Berg M. (2020). Surfactant Protein D Is Associated with Severe Pediatric ARDS, Prolonged Ventilation, and Death in Children with Acute Respiratory Failure. Chest.

[28] Lim A., Radujkovic A., Weigand M.A., Merle U. (2021). Soluble receptor for advanced glycation end products (sRAGE) as a biomarker of COVID-19 disease severity and indicator of the need for mechanical ventilation, ARDS and mortality. Ann. Intensiv. Care.

[29] Delgado C., Krötzsch E., Jiménez-Alvarez L.A., Ramírez-Martínez G., Márquez-García J.E., Cruz-Lagunas A., Morán J., Hernández C., Sierra-Vargas P., Avila-Moreno F. (2014). Serum surfactant protein D (SP-D) is a prognostic marker of poor outcome in patients with A/H1N1 virus infection. Lung.

[30] Klein E.Y., Monteforte B., Gupta A., Jiang W., May L., Hsieh Y., Dugas A. (2016). The frequency of influenza and bacterial coinfection: A systematic review and meta-analysis. Influenza Other Respir. Viruses.

[31] Park J., Pabon M., Choi A.M.K., Siempos I.I., Fredenburgh L.E., Baron R.M., Jeon K., Chung C.R., Yang J.H., Park C.-M. (2017). Plasma surfactant protein-D as a diagnostic biomarker for acute respiratory distress syndrome: Validation in US and Korean cohorts. BMC Pulm. Med..

[32] van Zoelen M.A., van der Sluijs K.F., Achouiti A., Florquin S., Braun-Pater J.M., Yang H., Nawroth P.P., Tracey K.J., Bierhaus A., van der Poll T. (2009). Receptor for advanced glycation end products is detrimental during influenza A virus pneumonia. Virology.

[33] White M.R., Crouch E., Vesona J., Tacken P.J., Batenburg J.J., Leth-Larsen R., Holmskov U., Hartshorn K.L. (2005). Respiratory innate immune proteins differentially modulate the neutrophil respiratory burst response to influenza A virus. Am. J. Physiol. Cell. Mol. Physiol..

[34] Oczypok E.A., Perkins T.N., Oury T.D. (2017). All the “RAGE” in lung disease: The receptor for advanced glycation endproducts (RAGE) is a major mediator of pulmonary inflammatory responses. Paediatr. Respir. Rev..

[35] Tsai Y., Chiang K., Hung J., Chang W., Lin H., Shieh J., Chong I., Hsu Y. (2018). Der f1 induces pyroptosis in human bronchial epithelia via the NLRP3 inflammasome. Int. J. Mol. Med..

[36] Latz E., Xiao T.S., Stutz A. (2013). Activation and regulation of the inflammasomes. Nat. Rev. Immunol..

[37] Major J., Crotta S., Llorian M., McCabe T.M., Gad H.H., Priestnall S.L., Hartmann R., Wack A. (2020). Type I and III interferons disrupt lung epithelial repair during recovery from viral infection. Science.

[38] Robinson K.M., Choi S.M., McHugh K.J., Mandalapu S., Enelow R.I., Kolls J.K., Alcorn J.F. (2013). Influenza A Exacerbates Staphylococcus aureus Pneumonia by Attenuating IL-1β Production in Mice. J. Immunol..

[39] Ataide M.A., Andrade W.A., Zamboni D.S., Wang D., Souza M.D.C., Franklin B.S., Elian S., Martins F.S., Pereira D., Reed G. (2014). Malaria-Induced NLRP12/NLRP3-Dependent Caspase-1 Activation Mediates Inflammation and Hypersensitivity to Bacterial Superinfection. PLoS Pathog..

[40] Buszko M., Park J.-H., Verthelyi D., Sen R., Young H.A., Rosenberg A.S. (2020). The dynamic changes in cytokine responses in COVID-19: A snapshot of the current state of knowledge. Nat. Immunol..

[41] Kudo S., Mizuno K., Hirai Y., Shimizu T. (1990). Clearance and tissue distribution of recombinant human interleukin 1 beta in rats. Cancer Res..

[42] Dinarello C.A. (2011). Interleukin-1 in the pathogenesis and treatment of inflammatory diseases. Blood.

[43] Pascual V., Allantaz F., Arce E., Punaro M., Banchereau J. (2005). Role of interleukin-1 (IL-1) in the pathogenesis of systemic onset juvenile idiopathic arthritis and clinical response to IL-1 blockade. J. Exp. Med..

[44] Merad M., Martin J.C. (2020). Pathological inflammation in patients with COVID-19: A key role for monocytes and macrophages. Nat. Rev. Immunol..

[45] Vora S.M., Lieberman J., Wu H. (2021). Inflammasome activation at the crux of severe COVID-19. Nat. Rev. Immunol..

[46] Sefik E., Qu R., Junqueira C., Kaffe E., Mirza H., Zhao J., Brewer J.R., Han A., Steach H.R., Israelow B. (2022). Inflammasome activation in infected macrophages drives COVID-19 pathology. Nature.

[47] Stanzel F. (2012). Bronchoalveolar Lavage. Principles and Practice of Interventional Pulmonology.

[48] Salina A., Schumann D.M., Franchetti L., Jahn K., Purkabiri K., Müller R., Strobel W., Khanna N., Tamm M., Stolz D. (2022). Multiplex bacterial PCR in the bronchoalveolar lavage fluid of non-intubated patients with suspected pulmonary infection: A quasi-experimental study. ERJ Open Res..

