# Supplementary material for: Increased Alveolar Epithelial Damage Markers and Inflammasome-Regulated Cytokines Are Associated with Pulmonary Superinfection in ARDS
Source: J Clin Med. 2023 May 24;12(11):3649. doi: 10.3390/jcm12113649 (PMC10253810; doi:10.3390/jcm12113649)
Supplement: Supplementary file 1 [file jcm-12-03649-s001.zip › jcm-2313727-supplementary.pdf]

Supplementary Table S1. Origin of ARDS in patients with and without pulmonary superinfection

Patient	Sex	Age	Superinfection	Origin of ARDS
1	male	58	yes	viral
2	male	51	no	viral
3	male	58	no	viral
4	female	43	yes	viral
5	male	45	yes	viral
6	female	56	yes	viral
7	female	77	yes	viral
8	male	70	yes	viral
9	male	63	yes	viral
10	male	26	yes	bacterial
11	male	74	no	bacterial
12	male	63	yes	other
13	male	62	yes	bacterial
14	male	78	yes	bacterial
15	male	56	yes	viral
16	female	68	no	bacterial
17	male	43	no	non-pulmonary
18	female	40	no	non-pulmonary
19	male	58	no	non-pulmonary
20	male	52	yes	non-pulmonary
21	male	73	yes	bacterial
22	male	69	no	bacterial
23	male	26	no	non-pulmonary
24	male	60	yes	bacterial
25	male	36	yes	bacterial
26	female	54	no	bacterial
27	female	48	no	bacterial
28	female	70	yes	bacterial
29	male	57	no	viral
30	male	53	no	viral
31	male	27	yes	viral
32	male	63	no	viral
33	male	48	no	viral
34	female	48	no	viral
35	male	46	no	non-pulmonary
36	male	76	no	bacterial
37	male	51	no	non-pulmonary
38	male	62	yes	viral
39	female	55	yes	viral
40	male	69	yes	viral
41	female	33	yes	viral
42	female	45	yes	viral
43	female	69	yes	viral
44	male	44	no	bacterial
45	male	55	no	non-pulmonary
46	male	43	no	non-pulmonary
47	male	55	no	non-pulmonary
48	male	58	no	bacterial
49	male	23	no	bacterial
50	male	34	no	bacterial
51	male	58	no	bacterial
52	male	67	no	bacterial
